# Arabidopsis Plastid-RNA Polymerase RPOTp Is Involved in Abiotic Stress Tolerance

**DOI:** 10.3390/plants9070834

**Published:** 2020-07-02

**Authors:** Abel Lidón-Soto, Eva Núñez-Delegido, Iván Pastor-Martínez, Pedro Robles, Víctor Quesada

**Affiliations:** Instituto de Bioingeniería, Universidad Miguel Hernández, Campus de Elche, 03202 Elche, Spain; abel.lidon@goumh.umh.es (A.L.-S.); eva.nunez@goumh.umh.es (E.N.-D.); ivan.pastor@goumh.umh.es (I.P.-M.); probles@umh.es (P.R.)

**Keywords:** Arabidopsis, RPOTp, salt stress, abiotic stress, plastid gene expression, abscisic acid

## Abstract

Plastid gene expression (PGE) must adequately respond to changes in both development and environmental cues. The transcriptional machinery of plastids in land plants is far more complex than that of prokaryotes. Two types of DNA-dependent RNA polymerases transcribe the plastid genome: a multimeric plastid-encoded polymerase (PEP), and a monomeric nuclear-encoded polymerase (NEP). A single NEP in monocots (RPOTp, RNA polymerase of the T3/T7 phage-type) and two NEPs in dicots (plastid-targeted RPOTp, and plastid- and mitochondrial-targeted RPOTmp) have been hitherto identified. To unravel the role of PGE in plant responses to abiotic stress, we investigated if Arabidopsis RPOTp could function in plant salt tolerance. To this end, we studied the sensitivity of T-DNA mutants *scabra3-2* (*sca3-2*) and *sca3-3*, defective in the *RPOTp* gene, to salinity, osmotic stress and the phytohormone abscisic acid (ABA) required for plants to adapt to abiotic stress. *sca3* mutants were hypersensitive to NaCl, mannitol and ABA during germination and seedling establishment. Later in development, *sca3* plants displayed reduced sensitivity to salt stress. A gene ontology (GO) analysis of the nuclear genes differentially expressed in the *sca3-2* mutant (301) revealed that many significantly enriched GO terms were related to chloroplast function, and also to the response to several abiotic stresses. By quantitative RT-PCR (qRT-PCR), we found that genes *LHCB1* (*LIGHT-HARVESTING CHLOROPHYLL a/b-BINDING*1) and *AOX1A* (*ALTERNATIVE OXIDASE 1A*) were respectively down- and up-regulated in the Columbia-0 (Col-0) salt-stressed plants, which suggests the activation of plastid and mitochondria-to-nucleus retrograde signaling. The transcript levels of genes *RPOTp*, *RPOTmp* and *RPOTm* significantly increased in these salt-stressed seedlings, but this enhanced expression did not lead to the up-regulation of the plastid genes solely transcribed by NEP. Similar to salinity, carotenoid inhibitor norflurazon (NF) also enhanced the *RPOTp* transcript levels in Col-0 seedlings. This shows that besides salinity, inhibition of chloroplast biogenesis also induces *RPOTp* expression. Unlike salt and NF, the NEP genes were significantly down-regulated in the Col-0 seedlings grown in ABA-supplemented media. Together, our findings demonstrate that RPOTp functions in abiotic stress tolerance, and RPOTp is likely regulated positively by plastid-to-nucleus retrograde signaling, which is triggered when chloroplast functionality is perturbed by environmental stresses, e.g., salinity or NF. This suggests the existence of a compensatory mechanism, elicited by impaired chloroplast function. To our knowledge, this is the first study to suggest the role of a nuclear-encoded plastid-RNA polymerase in salt stress tolerance in plants.

## 1. Introduction

The hypothesis that chloroplasts descend by endosymbiosis from erstwhile free-living, photosynthetically active cyanobacteria is currently widely accepted by the scientific community [1]. Nearly all the genes of the initially genetically autonomous endosymbiont were lost or relocated to the nucleus of the host cell. As a result, its activity was finally dependent on the functions provided by the host [2]. However, the endosymbiont retained part of the ancestral DNA, which eventually gave rise to the genomes of the different types of currently-existing plastids, including chloroplasts. Compared to the ancestral cyanobacteria from which they evolved, chloroplasts contain a very small genome (plastome) that typically ranges from 120 to 160 kb in size, and harbors only 90 to 100 genes, mostly involved in photosynthesis and plastid gene expression (PGE; [3]). PGE regulation can occur via the modulation of the number of copies of the plastome, or through mechanisms of transcriptional, post-transcriptional, translational or post-translational control [4].

The transcriptional control of PGE is highly complex. Along this line, the transcriptional machinery of the chloroplasts of land plants is more complex than that of prokaryotes. Accordingly, plastid genes are transcribed by two types of DNA-dependent RNA polymerases: PEP (Plastid-Encoded Polymerase), a bacterial-type polymerase encoded by the plastome, and NEP (Nuclear-Encoded Polymerase), a monomeric-type polymerase similar to that of T3/T7 phages, encoded by the nuclear genome [4]. PEP is a multimeric enzyme composed of subunits α, β, β′ and β′′, which form the core of the enzyme (holoenzyme), and which are respectively encoded by plastome genes *rpoA*, *rpoB*, *rpoC1* and *rpoC2* [5,6,7]. When isolated from photosynthetically active plastids, the PEP holoenzyme is usually associated with several nuclear-encoded proteins, such as sigma factors (SIG), which confer promoter recognition, and the PEP-associated proteins (PAP) required for plastid transcriptional regulation [8,9,10,11]. In addition to PEP, the second level of the control of chloroplast transcription in land plants is represented by NEP polymerases. In monocots, a single NEP coded by the *RPOTp* gene (RNA polymerase T3/T7 phage-type) has been hitherto identified, whereas two NEPs are present in dicots: RPOTp, targeted to plastids, and RPOTmp, dually targeted to plastids and mitochondria [12,13,14,15]. Plastid RPOTp and RPOTmp polymerases most likely evolved from the duplication of an ancestral-nuclear gene encoding mitochondrial RPOTm polymerase [16]. Along these lines, the small *RPOT* gene family in Arabidopsis comprises three members, that encode mitochondrial- (RPOTm), chloroplast- (RPOTp) and both organelle-targeted (RPOTmp) polymerases [12,13,14].

In stark contrast to PEP, NEP polymerases are single catalytic subunits that perform the entire transcription process, from promoter recognition to termination [17]. PEP is considered the predominant plant-RNA polymerase in green organs because it transcribes most plastid genes [18]. Notwithstanding, NEP remains essential for the expression of the chloroplast genes *rpoB*, *rpoC1* and *rpoC2* (which constitute an operon), and *rpoA* and *accD* (which encode the β subunit of acetyl-CoA carboxylase for lipid biosynthesis), in dicots [4]. Despite the proposal that *RPOTp* and *RPOTmp* display their highest levels of activity in different tissues and/or developmental stages in dicots, very few data currently support a distribution of functions between both RNA polymerases. In line with this, Arabidopsis RPOTmp activity has been reported in the non-photosynthetically and actively dividing cells of different organs, while that of RPOTp has been detected mainly in cells of green tissues [19].

Plant mutant analysis suggests the fundamental role of both PEP and NEP in chloroplast transcription, and, hence, in this organelle’s biogenesis and function. Along these lines, tobacco PEP-defective mutants are impaired in photosynthesis, and display an albino phenotype [18,20,21]. Knocking out Arabidopsis genes *RPOTmp* [22], and principally *RPOTp*, the latter in *scabra3* (*sca3*) mutants [23], has resulted in abnormal chloroplast development, stunted growth, reduced pigmentation and altered leaf morphogenesis. Interestingly, the *rpoTmp sca3-2* double mutant individuals exhibit a more severe phenotype, and even arrest growth early in development [23].

Chloroplasts may play a fundamental role in plant stress responses by sensing abiotic stress and transmitting this information to the nucleus, in order to coordinate the expression of nuclear and plastome genes. In this way, organelle activity adjusts to the new environmental conditions, which allows plants to cope with stress [24]. Accordingly, PGE has to adequately respond to changes in development, and also to environmental stimuli. Accordingly, it has recently been reported that a disruption of the function of genes involved in PGE alters a plant’s response to abiotic stress [24,25]. In the last few years, several nuclear genes sensitive to salinity that encode plastid proteins have been described, and some are also involved in PGE regulation [26,27]. Besides, Arabidopsis mutants affected in nuclear genes required for PGE show altered responses to different abiotic stresses [24,25]. Notwithstanding, currently available information on the effects of altered PGE on plant stress tolerance is still limited.

To gain insight into the role of PGE in plant responses to abiotic stress, we decided to investigate whether NEP RPOTp could be involved in plant salt tolerance. Two strong loss-of-function mutant alleles of the *RPOTp* gene (*sca3-2* and *sca3-3*) were morphologically and molecularly characterized in a previous work [23]. However, whether the *SCA3* (*RPOTp*) gene plays a role in tolerance to abiotic stress remains to be assessed. We report herein a study of the sensitivity of *sca3* mutants to not only ionic and osmotic stresses, but also to the abscisic acid (ABA) hormone involved in plant responses to stress. We performed an analysis of gene ontologies (GO) for the genes differentially expressed in the *sca3-2* mutant, and investigated the effect of salt stress and ABA on the expression of NEP and plastome genes. We then examined *RPOTp* expression in response to carotenoid inhibitor norflurazon (NF). Our results reveal that RPOTp function, which is required for abiotic stress tolerance, is controlled by plastid-to-nucleus retrograde signaling, which is triggered when chloroplast functionality is perturbed by environmental stress. As far as we know, this is the first study to suggest a role for NEP in salt stress tolerance in plants.

## 2. Results

### 2.1. Knock-Down of RPOTp Alters NaCl, Mannitol and ABA Responses

To determine if plastid RNA polymerase RPOTp could be involved in plants’ tolerance to environmental stress, we studied the sensitivity of Arabidopsis wild-type Columbia-0 (Col-0) and T-DNA mutants *sca3-2* and *sca3-3* [23] to different abiotic stresses. To this end, Col-0 and *sca3* mutant seeds were sown in a culture media supplemented with 150 mM NaCl or 350 mM mannitol, which respectively cause ionic and osmotic stress, and their ability to germinate and to form fully expanded green cotyledons (seedling establishment) was examined for 14 DAS (days after stratification).

**Figure 1:**
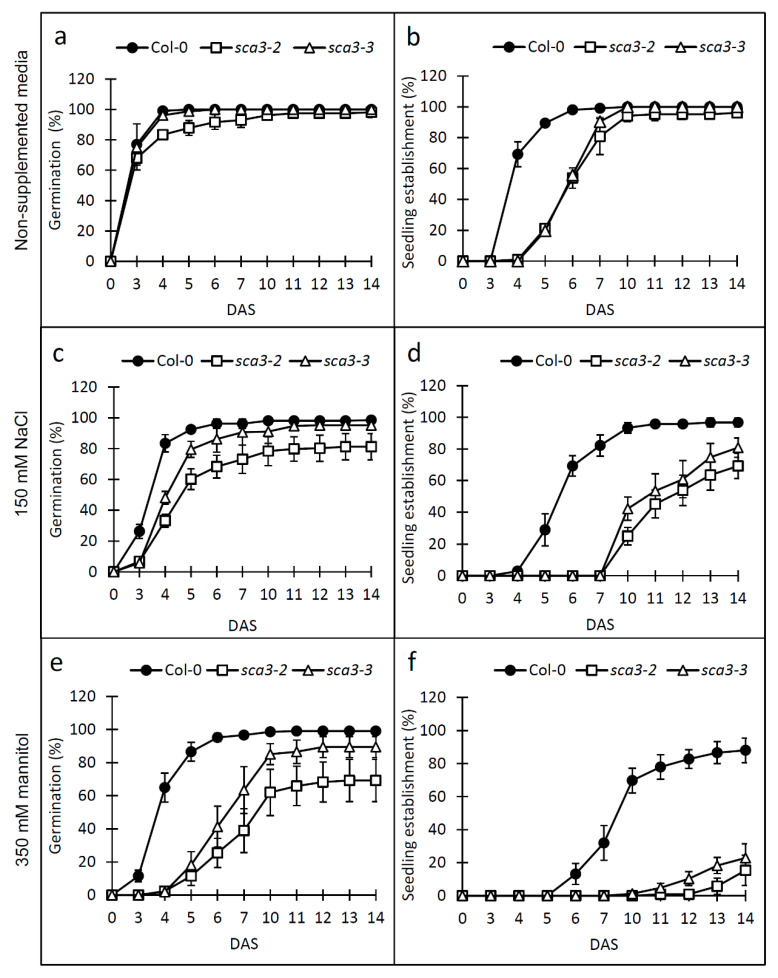
Effects of NaCl and mannitol on germination and seedling establishment in the wild-type Col-0 and mutants *sca3-2* and *sca3-3*. Each value corresponds to the mean ± standard deviation (SD) of the percentage of germination (**a**,**c**,**e**) and seedling establishment (**b**,**d**,**f**) in growth media, either without supplementation (**a**,**b**), or supplemented with 150 mM NaCl (**c**,**d**) or 350 mM mannitol (**e**,**f**), for four replicates of at least 50 seeds each per genotype. DAS: days after stratification.

In the absence of stress, the *sca3* mutant seeds yielded similar germination values to those of the wild-type seeds, although the *sca3-2* mutant showed slightly lower values than Col-0 during the first culture week (Figure 1a). NaCl or mannitol exogenously applied to the growth medium delayed Col-0 and *sca3* germination, although this effect was much more pronounced in both mutants, particularly in *sca3-2* (Figure 1c). In response to mannitol, the germination differences between Col-0 and the *sca3* mutants became even more evident, and the *sca3-2* mutant was more sensitive than *sca3-3* (Figure 1e).

Seedling establishment was delayed in the *sca3* mutants compared to Col-0 for the first 7 DAS in the MS control medium (Figure 1b). This is consistent with the defective photoautotrophic growth previously reported for these mutants [23]. The differences between Col-0 and the *sca3* mutants were substantially marked in the presence of 150 mM NaCl or 350 mM mannitol, and *sca3-2* was more sensitive than *sca3-3* (Figure 1d,f). In line with this, at 10 DAS, 95%, 42% and 25% seedling establishment was achieved for the Col-0, *sca3-3* and *sca3-2* mutant seeds, respectively, with 150 mM of NaCl (Figure 1d). Furthermore, at 14 DAS in the presence of mannitol, 90% of the Col-0 seeds yielded seedlings with green expanded cotyledons, as opposed to only 15% and 23% of *sca3-2* and *sca3-3*, respectively (Figure 1f).

**Figure 2:**
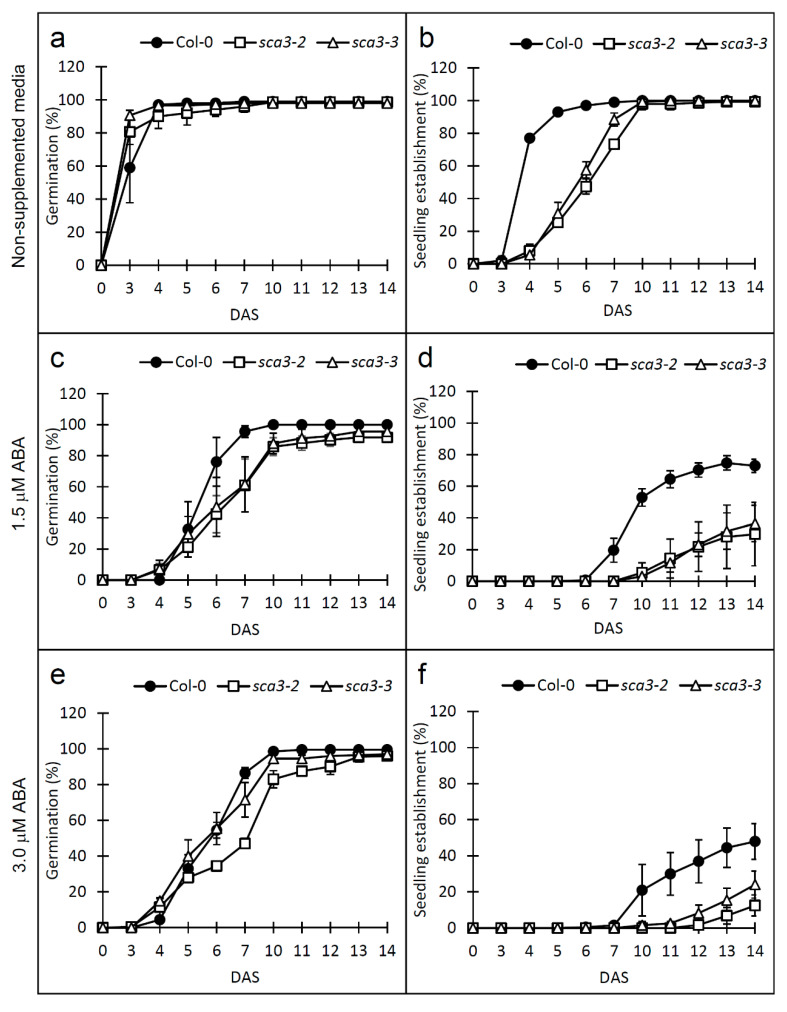
Effects of ABA on germination and seedling establishment in the wild-type Col-0 and mutants *sca3-2* and *sca3-3*. Each value corresponds to the mean ± standard deviation (SD) of the percentage of germination (**a**,**c**,**e**) and seedling establishment (**b**,**d**,**f**) in growth media, either without supplementation (**a**,**b**), or supplemented with 1.5 µM ABA (**c**,**d**) or 3 µM ABA (**e**,**f**), for four replicates of at least 50 seeds each per genotype.

The ABA hormone is induced in response to abiotic stress, and plays a fundamental role in adapting plants to adverse environmental conditions [28]. Mutants displaying altered sensitivity to abiotic stress frequently exhibit abnormal responses to ABA [25]. The enhanced sensitivity of the *sca3* mutants to NaCl and mannitol prompted us to investigate their response to ABA. To do so, the *sca3* and Col-0 seeds were sown in the MS growth media supplemented with 1.5 or 3 µM ABA. Germination of the *sca3* mutants was more sensitive to ABA than that of Col-0, especially *sca3-2* in the presence of 3 µM ABA (Figure 2a,b), which is similar to the results obtained with salt or mannitol stress (Figure 1). These differences were more marked from 6 to 10 DAS (Figure 2a,c,e). The ABA concentrations led to a noticeable reduction in seedling establishment in the Col-0 and *sca3* mutants throughout the study period, although the mutants were more sensitive than Col-0. Accordingly, at 14 DAS, 73% of the Col-0 seeds, and 30% and 37% of the *sca3-2* and *sca3-3* seeds, respectively, yielded seedlings with green expanded cotyledons, with 1.5 µM ABA (Figure 2d). These percentages lowered to 48%, 12% and 24% for Col-0, *sca3-2* and *sca3-3*, respectively, with 3 µM ABA (Figure 2f).

To confirm the role of RPOTp in abiotic stress tolerance, we also calculated, from 0 to 14 DAS, the Col-0/*sca3-2* and Col-0/*sca3-3* ratios for both percentages of germination and seedling establishment, under control conditions and in response to 150 mM NaCl, 350 mM mannitol, or 1.5 or 3 µM ABA (Table S1). In non-supplemented growth media, the Col-0/*sca3-2* and Col-0/*sca3-3* values were very similar (usually 1, or very close to 1). In the presence of NaCl, mannitol or ABA, the Col-0/*sca3* ratios for germination and seedling establishment yielded higher values than they did in non-supplemented growth media, which is in line with the hypersensitivity of *sca3* mutants to these stress conditions (Table S1; Figure 1 and Figure 2). Interestingly, the Col-0/*sca3-2* ratios were usually higher than those of Col-0/*sca3-3*, which is consistent with the enhanced sensitivity of *sca3-2* to stress (Table S1).

Taken together, our results showed that the *sca3* mutants were hypersensitive to not only ionic and osmotic stresses, but also to ABA during germination and seedling establishment, and *sca3-2* was more sensitive than *sca3-3*.

**Figure 3:**
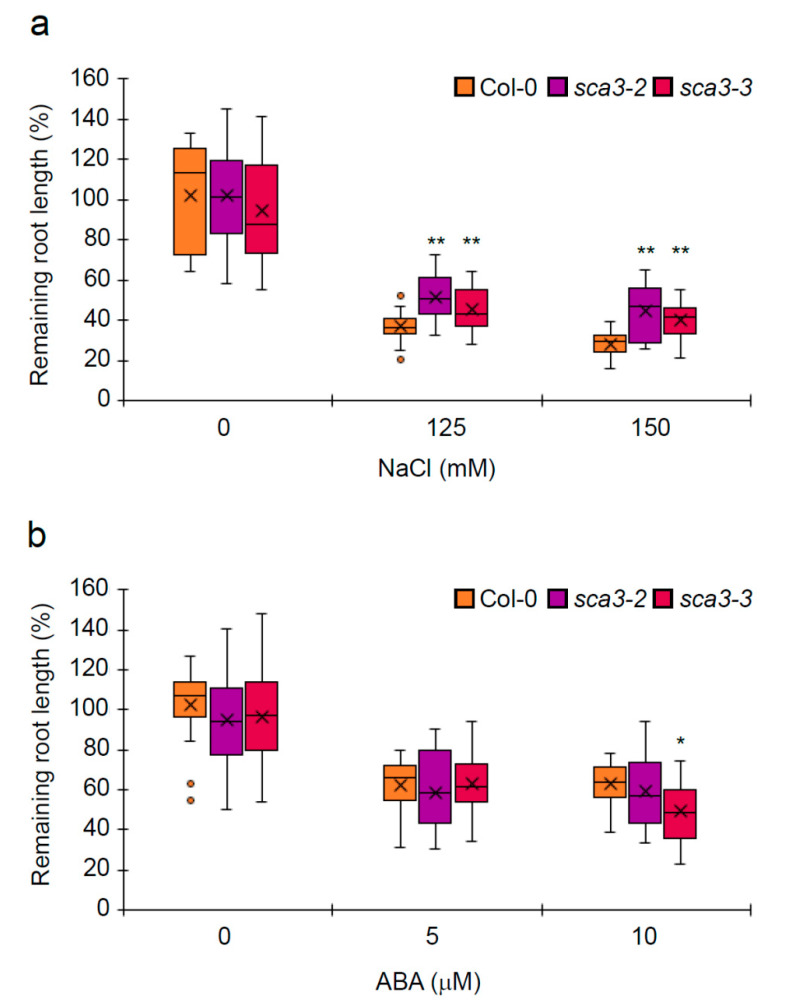
Box plots for sensitivity to ABA and NaCl of *sca3* plants. Individuals were transplanted at 7 DAS from the non-supplemented growth media to the media supplemented with either 125 or 150 mM NaCl, or 5 or 10 µM ABA. Seven days after the transfer, the root lengths of the plants transferred to the NaCl- (**a**) or ABA- (**b**) supplemented media were determined and referred to those of the same genotypes transferred to the non-supplemented media. These values are represented as percentages of root length of the plants transferred to the non-supplemented media. Dark horizontal lines represent the median, (×) denotes the mean, the box represents the 25th and 75th percentiles, whiskers represent the 1.5 IQR (Inter-Quartile Range) limits and outliers are shown by dots (n ≥ 15). Values significantly differed from Col-0 at * *p* < 0.05 or ** *p* < 0.01, according to a Student’s t-test.

We also evaluated the response of the *sca3* plants to salt and ABA later in development, without the detrimental effect that NaCl and ABA exert on germination and seedling establishment. To this end, Col-0 and mutant plants were transferred, at 7 DAS, from non-supplemented agar medium to MS-control media, or media supplemented with NaCl (125 or 150 mM NaCl) or ABA (5 or 10 μM). Root length was determined at 14 DAS (see Materials and Methods). The roots of the *sca3* individuals were significantly shorter than those of the Col-0, in the MS-control medium or in the presence of NaCl or ABA (Figure S1). With salt stress, the *sca3* mutants were significantly less sensitive than Col-0 to the root growth inhibition caused by either 125 mM or 150 mM NaCl (Figure 3a). With the ABA-supplemented media, no significant differences in root length inhibition were found between the *sca3* and Col-0 plants, except for the *sca3-3* mutant in response to 10 μM ABA, which was slightly more sensitive than Col-0 (Figure 3b).

### 2.2. Analysis of Gene Ontologies in the Transcriptome of the sca3-2 Mutant

A microarray analysis of the transcriptome of the *sca3-2* mutant, previously performed using total RNA extracted from 3-week-old plants, revealed 301 differentially expressed (DE) nuclear genes, 198 and 103 down- and up-regulated, respectively, compared to Col-0 [23]. No organellar genes were present in the array. We decided to examine the set of DE in *sca3-2* to identify functions related to abiotic stress response. To this end, we carried out an analysis of gene ontologies (GO) using the Singular Enrichment Analysis (SEA) online tool, accessed through agriGO v2.0 [29]. This allowed us to find which GO terms are enriched, that is, over-represented (or under-represented), using annotations for a given gene set. Enriched GO terms appear, statistically, more (or less) frequently than would be expected just by chance when analyzing the list of annotated terms for the set of input genes.

We performed the GO enrichment analysis on the sets of 198 under-expressed and 103 over-expressed genes separately. For the under-expressed genes, we found 95 terms significantly enriched (all of them over-represented), of which 48, 38 and 9 terms belonged to the ‘biological process’, ‘cellular component’ and ‘molecular function’ sub-ontologies, respectively (Table S2). Interestingly, we identified numerous GO terms that were associated with environmental stress, and which were significantly enriched in the set of under-expressed genes in the *sca3-2* mutant. The most significantly enriched term in the ‘biological process’ was ‘response to a low light intensity stimulus’ (GO:0009645), as 26.35% of the genes containing this term in the background set of genes were DE in *sca3-2* (Table S2). Interestingly, most of the identified terms in this sub-ontology were associated with the response to external stimuli, with different types of stresses: ‘response to water deprivation’ (GO:0009414), ‘response to oxidative stress’ (GO:0006979), ‘response to abiotic stimulus’ (GO:0009628), ‘response to cold’ (GO:0009409), ‘response to osmotic stress’ (GO:0006970) or ‘response to stress’ (GO:0006950). Another group of terms significantly enriched in this ontology were those related to photosynthesis, such as ‘photosynthesis, light harvesting’ (GO:0009765), ‘photorespiration’ (GO:0009853) or ‘photosynthesis, light reaction’ (GO:0019684) (Table S2). For the ‘molecular function’ sub-ontology, the most significantly enriched term was ‘pigment binding’ (GO:0031409), as 22.72% of the genes in the background set of genes containing this term were DE in *sca3-2*. Consistent with this, other significantly enriched terms were ‘chlorophyll binding’ (GO:0016168), ‘tetrapyrrole binding’ (GO:0046906) and ‘catalytic activity’ (GO:0003824) (Table S2). Finally, in relation to the ‘cellular component’ sub-ontology, it is worth noting the significantly enriched GO terms related to chloroplasts, and particularly to the thylakoid, which fell in line with the terms found in the other two sub-ontologies: ‘photosystem I’ (GO:0009522), ‘plastoglobule’ (GO:0010287), ‘chloroplast thylakoid membrane protein complex’ (GO:0098807), ‘thylakoid lumen’ (GO:0031977), ‘chloroplast thylakoid’ (GO:0009534), ‘organelle subcompartment’ (GO:0031984) or ‘plastid stroma’ (GO:0009532). ‘Light-harvesting complex’ (GO:0030076) was the most significantly enriched term (20% of all the genes containing this term in the background set of genes were under-expressed in *sca3-2*) (Table S2).

As regards the over-expressed genes, we found only eight terms significantly enriched, all of them over-represented and belonging to the ‘cellular component’ sub-ontology, but none of them related to the terms identified from the under-expressed genes (Table S2).

Together, these results indicate that many *sca3-2* down-regulated genes were involved in either photosynthesis or abiotic stress responses. Consequently, the diminished activity of these genes in this mutant could account for *sca3*-defective photoautotrophic growth [23] and the altered sensitivity to stress.

### 2.3. RPOTp Expression is Controlled by Plastid Retrograde Signaling in Response to Salt Stress

To gain further insight into the role of PGE in plant responses to abiotic stress, more specifically to salinity, we decided to investigate the effect that salt stress could have on the steady-state levels of the transcripts of the three Arabidopsis NEP genes: *RPOTp*, *RPOTmp* and *RPOTm*. We decided to perform this analysis on the Col-0 seedlings grown under similar conditions to those we previously assayed (see Figure 1, and Materials and Methods). To this end, Col-0 seedlings were grown in medium supplemented, or not, with 100 mM NaCl. This condition significantly delayed seedling growth, but did not impair growth as severely as 150 mM NaCl (the concentration we used in the germination and seedling establishment assays) (Figure 1). At 10 DAS, total RNA was extracted and retro-transcribed, and cDNAs were analyzed by quantitative PCR (qPCR). To confirm the effectiveness of the salt stress treatment, we first studied the expression of the *RD29A* (*RESPONSIVE TO DESICCATION 29A*) and *COR15B* (*COLD-REGULATED 15B*) genes, which are induced by exposure to salinity, cold or ABA [30]. As expected for salt stress, the transcript levels of *RD29A* and *COR15B* significantly increased in the Col-0 seedlings grown on 100 mM NaCl (4.8- and 2.7-fold up-regulated, respectively; Table 1). As regards the expression of the NEP genes, the treatment with 100 mM NaCl led to a significant increase in the steady-state levels of the transcripts of them all, albeit to different extents. Accordingly, *RPOTm*, *RPOTp* and *RPOTmp* were 1.3-, 2.3- and 3.5-fold up-regulated, respectively (Table 1). To determine the specificity of the effect of salt stress on the expression of the NEP genes, we studied the transcript levels of two nuclear genes that encode chloroplast-targeted proteins also involved in PGE: the mitochondrial transcription termination factors mTERF5 and mTERF9. These mTERFs are functionally related to RPOTp and are required for chloroplast development and response to abiotic stress [31]; furthermore, mTERF5 regulates transcription of the plastid *psbEFLJ* polycistron [32]. In stark contrast to the NEP genes, we found that 100 mM of NaCl significantly repressed *mTERF5* expression (1.3-fold down-regulated) and did not noticeably modify *mTERF9* transcript abundance (Table 1).

Together, our results reveal that salinity differentially affected the expression of nuclear genes involved in chloroplast transcription. To investigate if these changes in gene activity could result from retrograde signaling from chloroplasts to the nucleus, we decided to study, in the salt-stressed and non-salt stressed control Col-0 seedlings, the mRNA abundance of two marker genes for the activation of retrograde signaling from the chloroplast and mitochondria to the nucleus. To this end, the expression of the photosynthesis gene *LHCB1* (encoding the LIGHT-HARVESTING CHLOROPHYLL a/b-BINDING1 protein) and the *AOX1A* gene (encoding ALTERNATIVE OXIDASE 1A) were analyzed by qRT-PCR. It is well-known that the *LHCB1* gene is down-regulated when chloroplast biogenesis is perturbed [33], whereas *AOX1A* expression increases in response to impaired mitochondria function, in order to protect plants from oxidative damage and prevent reactive oxygen species (ROS) production [34,35]. Compared to the non-stressed seedlings, accumulation of the *LHCB1* transcripts significantly decreased (1.4-fold down-regulated), whereas *AOX1A* mRNAs markedly increased (3.9-fold up-regulated), in the seedlings exposed to 100 mM NaCl (Table 1). Therefore, these results indicated that our salt stress conditions could trigger organelle retrograde signaling, which would, in turn, modulate the expression of NEP genes.

**Figure 4:**
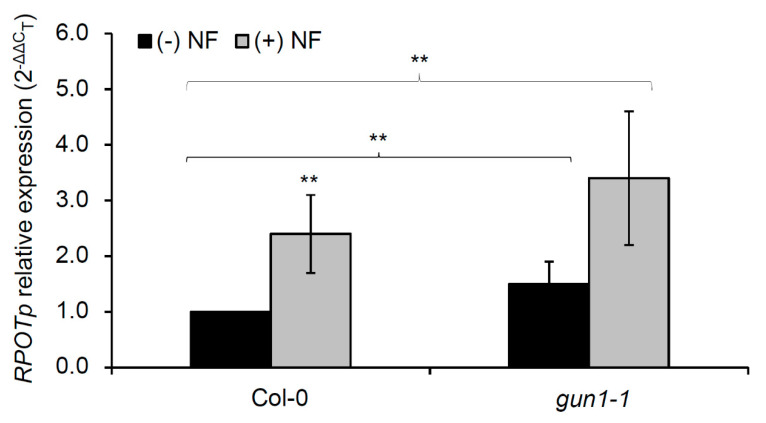
Analysis by qRT-PCR of *RPOTp* gene expression in Col-0 and the *gun1-1* mutant in response to norflurazon (NF). *RPOTp* expression in Col-0 and the *gun1-1* mutant in growth media supplemented (+) or not (−) with NF. Relative values were determined by the 2^−^^ΔΔ*C*T^ method for the *RPOTp* gene after normalization with those of the *ACTIN2* gene, and compared to those of Col-0 in the non-supplemented media, to which a value of 1 was assigned. Each value corresponds to the mean ± SD of three biological replicates and triplicate reactions. Asterisks indicate significant differences using a Mann–Whitney U-test (** *p* < 0.01) in the supplemented vs. non-supplemented media for each genotype. Plants were collected at 6 DAS.

To study the regulation of NEP function by impaired chloroplast biogenesis in-depth, we analyzed the expression of the *RPOTp* gene in Col-0 seedlings grown in the presence or absence of NF, a bleaching herbicide that inhibits carotenoid biosynthesis and, consequently, chloroplast functionality [36]. When seedlings are treated with NF, the expression of photosynthesis-associated nuclear genes (such as *LHCB1*) is repressed, which has been interpreted as a result of plastid-to-nucleus retrograde signaling [37,38,39]. In our study, we included the *gun1-1* mutant defective in the GUN1 protein, a central integrator of plastid retrograde signals [40]. Compared to the Col-0 seedlings grown on the control media, the presence of NF significantly increased *RPOTp* transcript abundance (2.4-fold up-regulated) (Figure 4), which was similar to 100 mM NaCl. In the absence of NF, *RPOTp* expression in the *gun1-1* mutant was significantly higher than in Col-0 (1.5-fold up-regulated) (Figure 4). This result is similar to the up-regulation of photosynthesis genes previously reported in this mutant [25,33]. Along these lines, the accumulation of *RPOTp* transcripts in the *gun1-1* seedlings was higher than in Col-0 (1.6-fold up-regulated) when both strains were exposed to NF (Figure 4).

Therefore, we conclude that applying NF or salt stress similarly increased *RPOTp* expression in the wild-type seedlings, and revealed a positive regulation of RPOTp transcript levels by retrograde signaling when chloroplast function is perturbed due to adverse environmental conditions.

### 2.4. Salt Stress Differentially Affects the Transcript Abundance of Plastid-Encoded Genes

To investigate the effect of salinity on the expression of the plastome, we studied, in Col-0 salt-stressed and non-stressed seedlings, the steady-state levels of the transcripts of the functionally different plastid genes transcribed by PEP or NEP RNA polymerases (Table 1). The selected genes encode proteins involved in photosynthesis (*psaA*, *psaB* and *psbA* encode Photosystem I and II reaction center proteins, respectively), protein degradation (*clpP* encodes an ATP-dependent protease), translation (*rps18* encodes ribosomal protein S18), lipid biosynthesis (*accD* encodes the Carboxyltransferase β subunit of Acetyl-CoA carboxylase) and transcription (*rpoA*, *rpoB* and *rpoC1* encode the α, β and β′ subunits of PEP, respectively) (Table 1). Compared to the non-stressed seedlings, the presence of 100 mM NaCl lowered the transcript levels of the majority of the studied genes (Table 1). This reduction was statistically significant for the *psbA*, *rpoA* and *rpoB* genes (1.7-, 1.6- and 1.6-fold down-regulated, respectively), whereas no significant changes in transcript abundance were found for the remaining genes, except for *clpP*, which was slightly up-regulated (Table 1). Consequently, although salt stress induced the expression of the *RPOTp* gene, this did not correlate with the mRNA levels of the plastid genes, which are fundamentally transcribed by RPOTp (*rpoA*, *rpoB*, *rpoC1* and *accD*; Table 1).

### 2.5. ABA Down-Regulates the Expression of NEP and Genes Encoding PEP Subunits

ABA plays a pivotal role in the response of plants to different abiotic stresses [41]. Hence, we decided to investigate if the expression of NEP genes could also be affected by ABA. To this end, we determined by qRT-PCR the steady-state levels of the transcripts of the *RPOTp*, *RPOTmp* and *RPOTm* genes in 10 DAS Col-0 seedlings grown in the presence or absence of 1.5 μM ABA. We included in this analysis the aforementioned nuclear genes *mTERF5* and *mTERF9*, as well as plastid genes *rpoA* and *rpoB*, as representatives of genes essentially transcribed by NEP. We selected 1.5 μM ABA because this concentration slows and decreases Col-0 cotyledon greening, but to a lesser extent than 3 μM ABA (Figure 2d,f), and it provided a sufficient number of seedlings to study the ABA effects at 10 DAS.

To confirm the effectiveness of the employed phytohormone concentration, we first analyzed the expression of the ABA-induced gene *RD29A*, as we did for NaCl stress (see above). In response to 1.5 μM ABA, *RD29A* was significantly up-regulated (32.7 ± 9.0; *p* = 4.11 × 10^−4^), which was consistent with the induction of *RD29A* mRNA by ABA [30], and besides, it confirmed the effectiveness of our treatment. We found that ABA repressed the expression of all the studied genes, although this down-regulation was statistically significant for *RPOTp* (0.62 ± 0.21; *p* = 0.001), *mTERF5* (0.59 ± 0.29; *p* = 0.001), *mTERF9* (0.44 ± 0.22; *p* = 2.42 × 10^−6^), *rpoA* (0.52 ± 0.18; *p* = 4.113 × 10^−4^) and *rpoB* (0.57 ± 0.23; *p* = 4.113 × 10^−4^), but not for *RPOTm* (0.85 ± 0.32; *p* = 0.223) or *RPOTmp* (0.86 ± 0.42; *p* = 0.223).

## 3. Discussion

This work investigated the role of Arabidopsis plastid-RNA polymerase RPOTp in tolerance to abiotic stress. To this end, the germination and seedling establishment of strong loss-of-function mutants *sca3-2* and *sca3-3*, affected in the *RPOTp* gene, were studied under different abiotic stress conditions. *sca3* mutants were hypersensitive to NaCl, mannitol and ABA during germination and seedling establishment, and *sca3-2* was more sensitive to stress than *sca3-3*. This is consistent with the location of the T-DNA insertions in the *RPOTp* gene in these mutants (intron 3 and exon 18 in *sca3-2* and *sca3-3*, respectively [23]). Along these lines, it has been reported that the *sca3-2* mutant exhibits enhanced sensitivity to cold in the seedling stage [23]. We consider that the hypersensitivity to stress displayed by the *sca3* mutants is due to impaired RPOTp function, and does not result from a delayed germination caused by the stress treatments. Consistent with this, other chloroplast-defective mutants, such as *mterf9* [which also shows, like *sca3* plants, stunted growth and impaired chloroplast biogenesis, and is morphologically very similar to *sca3* (both mutations even interact synergistically)], display enhanced tolerance to abiotic stress [31,42]. As such, these results suggest that *RPOTp* promotes tolerance to environmental stress during germination and seedling establishment. Later in development, RPOTp would act differently, because the *sca3-2* and *sca3-3* roots were less sensitive than Col-0 to NaCl. However, we cannot rule out that this deviating response to stress could be due to the different activities of RPOTp and RPOTmp in tissues and developmental stages [4], because *RPOTmp* and *RPOTp* expressions were detected mainly in the non-green cells of different organs and in photosynthetically active tissues, respectively [43]. Alternatively, the lesser reduction in root length of *sca3* plants exposed to salt stress, compared to the wild-type, might result from the *sca3-2* and *sca3-3* roots being closer to a growth limit situation (Figure S1).

Altered sensitivity to salt stress has been reported for other Arabidopsis mutants defective in genes involved in PGE [26,42,44,45]. Noteworthy among these genes, *mTERF5*, *mTERF6* and *SIGMA FACTOR5* (*SIG5*) regulate chloroplast transcription. Accordingly, mTERF5 acts as a PEP transcriptional pausing factor that specifically modulates the transcription of plastid *psbEFLJ* polycistron [32], whereas mTERF6 promotes the transcription termination of plastid *rpoA* polycistron [46]. As for *sca3* mutants, *sig5-2* germination is hypersensitive to salinity, and *SIG5* expression, unlike the remaining *SIG* genes, is induced in response to salinity and other abiotic stresses [47]. Nagashima et al. [47] suggested SIG5 is involved in protecting chloroplasts under abiotic stress by promoting the repair of the PSII reaction center. Recently, Zhao et al. [48] obtained transgenic lines overexpressing *SIG5*, which showed increased tolerance to salinity. As such, as these results show that both RPOTp- and PEP-mediated plastid transcription are involved in plant salt tolerance, we consider it feasible that PGE alteration at the transcriptional level may account for the abiotic stress phenotype of mutants *sca3*, *mterf* and *sig5-2*. Consistently with this, *sca3-2* synergistically interacts with mutations *mterf6-5* and *mterf9* [31,49].

Our enrichment analysis of GO terms, which used the complete sets of under- and over-expressed genes in the *sca3-2* mutant, revealed that most of the enriched GO terms belonged to the ‘biological processes’ and ‘cellular components’ sub-ontologies, and matched the expected function of RPOTp as a plastid-RNA polymerase. Accordingly, the GO terms related to photosynthesis or chloroplasts were significantly enriched in the set of down-regulated genes in *sca3-2* plants. Besides, the GO terms associated with the response to different abiotic stresses were also significantly over-represented in this set of under-expressed genes, which falls in line with the stress phenotypes of the *sca3-2* mutant. Consequently, the stress hypersensitivity of the *sca3-2* mutant might be due to the down-regulation of these genes, at least in part.

Chloroplast genome expression is under dual genetic control, and changes in the activity of plastid-RNA polymerases, due to endogenous or external cues, can seriously affect chloroplast function. As such, we studied the effect of continuous exposure to moderate salt stress or ABA on the expression of NEP genes. Our results showed that the transcript levels of NEP genes significantly increased in response to salinity, albeit to different extents, because the mRNAs of genes *RPOTmp* and *RPOTp* respectively accumulated nearly three- and twofold more than those of *RPOTm*. In line with this, Danilova et al. [50] reported a higher level of expression of the *RPOTmp* vs. *RPOTp* genes under heat stress in Arabidopsis. Interestingly, NEP up-regulation by salt stress was not found in genes *mTERF5* or *mTERF6*, which suggests a differential effect of salinity on the expression of the nuclear components of the plastid gene expression machinery. It is worth noting the enhanced expression of NEP genes in salt-stressed seedlings, which did not result in a significant up-regulation of plastid genes solely transcribed by NEP. This result somehow resembles the effect of heat stress on PEP and NEP expression, as reported by Danilova et al. [50]. These authors proposed heat stress causing PEP deficiency, which, in turn, would result in a compensatory increase in NEP expression at the mRNA level. Our findings fall in line with the activation of this compensatory mechanism—also in response to salinity—for maintaining chloroplast functionality. Along these lines, increased NEP transcript levels have been reported in mutants defective in PEP [21] or PAP [51] function. Reduced PEP activity by salt would account for the significant down-regulation of PEP-dependent PSII gene *psbA*, but it did not noticeably affect the expression of genes PSI *psaA* and *psaB*, also transcribed by PEP. A differential effect on the expression of plastid genes PSI and PSII has been reported in response to heat stress, which might be due to the different mechanisms governing the expression of these PS genes [50].

The effect of salinity on the accumulation and activity of NEP proteins remains to be elucidated. We have focused on the study of the steady-state levels of NEP mRNAs in response to salt stress. However, we cannot exclude that NEP up-regulation could be the consequence of impaired NEP activity by salinity, which still results in lower levels of NEP activity than under control conditions.

As transcripts of RPOTm, which is solely targeted to mitochondria, also accumulated in response to 100 mM NaCl, salinity also affected mitochondria activity. *RPOTm* up-regulation could be interpreted as an attempt to provide energy and to maintain metabolism under stress. Consistently with this, we found that the *AOX1A* gene was overexpressed in salt-grown seedlings, which agrees with the reported activation of the AOX pathway in response to impaired mitochondrial function [34].

We found that all the NEP and PEP genes herein investigated were down-regulated by the externally applied ABA. Hence the lower transcript levels of the NEP-dependent-plastid genes could be due to the lowered RPOTp activity in response to ABA. The inhibitory effect of ABA on the expression of chloroplast and NEP genes has been previously described. Accordingly, Yamburenko et al. [52] reported that exogenously applied ABA represses Arabidopsis plastid transcription by both PEP and NEP. Besides, ABA also reduces *RPOTp* transcript accumulation to about 50% of the control levels, whereas those of *RPOTmp* remain unaffected [52]. The same authors obtained similar results in barley leaves [53]. In line with this, Danilova et al. [54] found that most of the nuclear genes that encode different plastid transcriptional machinery components, including *RPOTp* and *RPOTmp*, were down-regulated in Arabidopsis seedlings exposed to ABA. This repressive effect of ABA on plastid gene expression could be mediated by alarmone guanosine-30-50-bisdiphosphate (ppGpp), which accumulates in chloroplasts in response to abiotic stress, and to ABA, jasmonate or ethylene hormones [55,56]. ppGpp binds to the β′-subunit of PEP, and inhibits the transcription that depends on this RNA polymerase. There is no evidence that ppGpp could repress RPOTp activity [56]. Therefore, *RPOTp* expression could be repressed by externally applying ABA through signaling to the nucleus, perhaps via PP2C and sucrose non fermenting protein-related kinases 2 (SnRK2) proteins, this being the pathway required for the expression of the enzymes that synthesize ppGpp [55]. Alternatively, *RPOTp* could be indirectly repressed by retrograde signal/s from chloroplasts to the nucleus, produced in response to ABA (e.g., ROS accumulating in response to reduced photosynthetic activity promoted by ABA [57]).

Our results show that the nuclear-photosynthesis gene *LHCB1*, a classic marker of plastid retrograde signaling, was down-regulated in salt-grown Col-0 seedlings. This falls in line with the reduced expression of Arabidopsis nuclear-photosynthetic genes reported by Štefanic et al. [58] under similar stress conditions. Therefore, these findings indicate that salinity could activate chloroplast-to-nucleus signaling, and repress *LHCB1*, which occurs in response to chloroplast biogenesis inhibitor NF. Along these lines, we found that *RPOTp* transcripts accumulated when chloroplast biogenesis was blocked by NF. Thus changes in *RPOTp* gene expression under salt stress, like that of *LHCB1*, may also be due to plastid-to-nucleus retrograde signaling, but likely take place through an ABA-independent mechanism, because *RPOTp* expression was not induced by ABA. Accordingly, osmotic stress can alter nuclear transcription via ABA-independent signaling pathways [59].

The perturbation of chloroplast development by mutations can also increase *RPOTp* expression. Accordingly, genes *RPOTp* and *RPOTm* were overexpressed in the leaves of the *albostrians* mutant of barley that lacks chloroplast translation [19]. We previously reported *RPOTp* up-regulation in Arabidopsis *mterf* mutants defective in chloroplast development [42,49,60,61]. Furthermore, the results by Schweer et al. [62] suggest that NEP function might be reactivated when PEP activity is altered by mutations.

The results discussed herein point out the existence of a rescue or compensatory mechanism triggered by impaired chloroplast function, which would lead to enhanced NEP transcript levels. GUN1 might be an important component of the NEP-dependent compensatory mechanism, because Tadini et al. [63] recently found that GUN1 physically interacts with RPOTp and promotes its activity when PEP function is impaired. Our results, and those of Danilova et al. [50], suggest that such a mechanism would also act in wild-type plants to help them cope with adverse environmental stimuli, like salt and heat stress. Notwithstanding, further research is required to better understand the mechanisms involved in reprogramming nuclear and plastid gene expressions in order to adapt chloroplast function and plant activity to salinity and other abiotic stresses.

## 4. Materials and Methods

### 4.1. Plant Materials and Growth Conditions

Plant cultures and crosses were carried out as reported in [61]. The seeds of *Arabidopsis thaliana* (L.) Heynh. wild-type accession Columbia-0 (Col-0) were obtained from the Nottingham Arabidopsis Stock Centre (NASC). The seeds of T-DNA insertional mutants *sca3-2* and *sca3-3* [23] and EMS-induced mutant *gun1-1* [64], all in the Col-0 genetic background, have been previously described. The *gun1-1* seeds were kindly provided by Dr. José Luis Micol.

### 4.2. Germination and Growth Sensitivity Assays

For the germination assays, sowings were performed as described in [61] on Petri dishes filled with GM agar medium [Murashige and Skoog (MS) medium containing 1% sucrose], supplemented with NaCl (150 mM), mannitol (350 mM) or ABA (1.5 or 3 µM). The seeds in which radicle emergence was observed were considered germinated, whereas seedling establishment was determined as the seedlings exhibiting green and fully expanded cotyledons. Seed germination and seedling establishment were scored from 1 to 14 DAS (days after stratification) on Petri dishes kept at 20 ± 1 °C, with 25 µmol·m^−2^·s^−1^ of photons.

For the NF treatment, seedlings were grown on Petri dishes filled with GM agar medium supplemented with 5 μM NF (Sigma-Aldrich, St. Louis, Missouri, USA).

To determine the salt and ABA responses during vegetative growth after seedling establishment, seeds were sown on non-supplemented GM agar medium, and seedlings were transferred at 7 DAS to new Petri dishes supplemented with NaCl (125 or 150 mM) or ABA (5 or 10 µM), and were vertically grown. Plant root length was determined after 7 days of NaCl or ABA treatment, to evaluate their tolerance to these stress conditions, by referring the values to those of the individuals transferred to the control (non-supplemented) media.

### 4.3. Quantitative RT-PCR (qRT-PCR)

Total RNA was extracted from 80 mg of wild-type Col-0 individuals at 6 and 10 DAS, and *gun1-1* mutant seedlings at 6 DAS, which were grown on GM agar medium not supplemented or supplemented with 100 mM NaCl or 5 μM NF. RNA was resuspended in 40 μL of RNase-free water and DNA was removed with the TURBO DNAfree kit (Invitrogen, Carlsbad, California, USA) following the manufacturer’s instructions. The cDNA preparations and qPCR amplifications were carried out in an ABI PRISM 7000 Sequence Detection System (Applied Biosystems, Foster City, California, USA) as described in [61] using the oligonucleotides listed in Table S1. Each reaction mix (20 μL) contained 7.5 μL of the SYBR-Green/ROX qPCR Master Kit (Fermentas, Vilnius, Lithuania), 0.4 μM of primers and 1 μL of cDNA solution. The relative quantification of gene expression data was performed by the 2^−ΔΔ*C*T^ or comparative C_t_ method as reported in [65]. Each reaction was done in three replicates, and three different biological replicates were used. The expression levels were normalized to the CT values obtained for the housekeeping *ACTIN2* gene [66].

### 4.4. Statistical Analyses

Statistical analyses were performed to compare, with the Col-0 non-stressed seedlings, the phenotypic traits and the relative transcript levels of the genes analyzed by qRT-PCR in the Col-0 seedlings treated with 100 mM NaCl, 1.5 μM ABA or 5 μM NF. Depending on the number of samples, a Mann–Whitney U-test (n ≤ 10) or a Student’s t-test (n > 10) was applied.

## 5. Conclusions

The present study demonstrates the role of plastid-RNA polymerase RPOTp in plant salt tolerance. We draw this conclusion from the analysis of the stress phenotypes of *RPOTp*-deficient mutants, and by studying the expression of NEP and plastid genes in Col-0 seedlings exposed to salt stress, ABA or NF. Our findings also show that plastid-to-nucleus retrograde signaling modulates *RPOTp* transcript levels, which supports the existence of a transcriptional compensatory mechanism triggered by impaired chloroplast function upon abiotic stress.

## Figures and Tables

**Table 1:** Quantitative RT-PCR analysis of the expression of chloroplast and nuclear genes in Col-0 seedlings exposed to 100 mM NaCl.

Gene	Normalized Transcript Levels in Col-0 Plants Exposed to 100 mM NaCl Relative to Control Plants		
	Protein Product	Fold Change	*p*-Value
*Nuclear genes*			
*RPOTp/SCA3*	NEP ^1^ RPOTp	2.27 ± 0.84	4.113 × 10^−5^ **
*RPOTmp*	NEP ^1^ RPOTmp	3.50 ± 1.70	3.996 × 10^−4^ **
*RPOTm*	NEP ^1^ RPOTm	1.34 ± 0.33	0.036 *
*AOX1A*	Alternative oxidase 1A	3.90 ± 1.00	4.113 × 10^−5^ **
*LHCB1*	Light-harvesting complex protein B1	0.70 ± 0.44	0.036 *
*mTERF5*	mTERF5	0.77 ± 0.31	0.008 **
*mTERF9*	mTERF9	1.10 ± 0.30	0.015 *
*COR15B*	Cold-regulated 15B	4.76 ± 2.11	2.20 × 10^−10^ **
*RD29A*	Responsive to desiccation 29A	2.74 ± 0.76	5.83 × 10^−4^ **
*Chloroplast genes*			
*psaA*	Photosystem I reaction center protein	1.12 ± 0.49	0.709
*psaB*	Photosystem I reaction center protein	1.04 ± 0.28	0.709
*psbA*	Chlorophyll binding protein D1	0.60 ± 0.22	4.113 × 10^−5^ **
*clpP*	ATP-dependent protease	1.18 ± 0.27	0.036 *
*rps18*	Ribosomal protein S18	0.83 ± 0.23	0.709
*rpoA*	PEP ^2^ α subunit	0.63 ± 0.27	0.001 **
*rpoB*	PEP ^2^ β subunit	0.61 ± 0.09	0.002 **
*rpoC1*	PEP ^2^ β’ subunit	0.86 ± 0.20	0.220
*accD*	Carboxytransferase β subunit of the Acetyl-CoA carboxylase	0.88 ± 0.35	0.709

^1^ NEP: nuclear-encoded plastid RNA polymerase. ^2^ PEP: plastid-encoded RNA polymerase. Relative expression values were determined as 2^−ΔΔ*C*T^ for each studied gene in 10 DAS Col-0 seedlings exposed to 100 mM after normalization with those of the *ACTIN2* gene, and compared with those of the Col-0 seedlings in control medium, to which a value of 1 was given (see Materials and Methods). Each value corresponds to the mean ± standard deviation of the 2^−ΔΔ*C*T^ values obtained using three different biological replicates and triplicate reactions. Values were significantly different from the corresponding wild type at *p* < 0.05 (*) or *p* < 0.01 (**) using Wilcoxon–Mann–Whitney test.

## Supplementary Materials

The following are available online at https://www.mdpi.com/2223-7747/9/7/834/s1, Table S1: Col-0/*sca3* ratios of germination and seedling establishment values under control and stress conditions. Table S2: Analysis of gene ontologies (GO) in the nuclear transcriptome of mutant *sca3-2*. Table S3: Primers used in this work. Figure S1: Box plots for the root length of *sca3* plants in response to ABA and NaCl. Individuals were transplanted at 7 DAS from the non-supplemented growth media to the media supplemented with either 125 or 150 mM NaCl, or 5 or 10 µM ABA. Seven days after transfer, the root lengths of the plants transferred to the NaCl- (A) or ABA- (B) supplemented media were determined. Dark horizontal lines represent the median, (×) denotes the mean, the box represents the 25th and 75th percentiles, whiskers represent the 1.5 IQR limits and outliers are shown by dots (n ≥ 15). Values significantly differed from the Col-0 at ** *p* < 0.01 according to a Student’s t-test.
