# Supplementary material for: Arabidopsis Plastid-RNA Polymerase RPOTp Is Involved in Abiotic Stress Tolerance
Source: Plants (Basel). 2020 Jul 2;9(7):834. doi: 10.3390/plants9070834 (PMC7412009; doi:10.3390/plants9070834)
Supplement: Supplementary file 1 [file plants-09-00834-s001.zip › plants-847469 supplementary/Table 1R FINAL.docx]

**Table 1.** Quantitative RT-PCR analysis of the expression of chloroplast and nuclear genes in Col-0 seedlings exposed to 100 mM NaCl

| Gene | Normalized transcript levels in Col-0 plants exposed to 100 mM NaCl relative to control plants | | | |
| --- | --- | --- | --- | --- |
|  | Protein product | Fold change | | p-value |
| *Nuclear genes* |  | |  |  |
| *RPOTp/SCA3* | NEP^1^ RPOTp | | 2.27 ± 0.84 | 4.113E-5^**^ |
| *RPOTmp* | NEP^1^ RPOTmp | | 3.50 ± 1.70 | 3.996E-4** |
| *RPOTm* | NEP^1^ RPOTm | | 1.34 ± 0.33 | 0.036* |
| *AOX1A* | Alternative oxidase 1A | | 3.90 ± 1.00 | 4.113E-5** |
| *LHCB1* | Light harvesting complex protein B1 | | 0.70 ± 0.44 | 0.036* |
| *mTERF5* | mTERF5 | | 0.77 ± 0.31 | 0.008** |
| *mTERF9* | mTERF9 | | 1.10 ± 0.30 | 0.015* |
| *COR15B* | Cold-regulated 15B | | 4.76 ± 2.11 | 2,20E-10** |
| *RD29A* | Responsive to desiccation 29A | | 2.74 ± 0.76 | 5,83E-04** |
| *Chloroplast genes* |  |  | |  |
| *psaA* | Photosystem I reaction center protein | 1.12 ± 0.49 | | 0.709 |
| *psaB* | Photosystem I reaction center protein | 1.04 ± 0.28 | | 0.709 |
| *psbA* | Chlorophyll binding protein D1 | 0.60 ± 0.22 | | 4.113E-5** |
| *clpP* | ATP-dependent protease | 1.18 ± 0.27 | | 0.036* |
| *rps18* | Ribosomal protein S18 | 0.83 ± 0.23 | | 0.709 |
| *rpoA* | PEP^2^ α subunit | 0.63 ± 0.27 | | 0.001** |
| *rpoB* | PEP^2^ β subunit | 0.61 ± 0.09 | | 0.002** |
| *rpoC1* | PEP^2^ β’ subunit | 0.86 ± 0.20 | | 0.22 |
| *accD* | Carboxytransferase β subunit of the Acetyl-CoA carboxylase | 0.88 ± 0.35 | | 0.709 |

^1^NEP: nuclear-encoded plastid RNA polymerase. ^2^PEP: plastid-encoded RNA polymerase. Relative expression values were determined as 2^-ΔΔCT^ for each studied gene in 10 DAS Col-0 seedlings exposed to 100 mM after normalization with those of the *ACTIN2* gene, and compared with those of the Col-0 seedlings in control medium, to which a value of 1 was given (see Materials and Methods). Each value corresponds to the mean ± standard deviation of the 2^-ΔΔCT^ values obtained using three different biological replicates and triplicate reactions. Values were significantly different from the corresponding wild type at P<0.05 (*) o P<0.01 (**) using Wilcoxon-Mann-Whitney test.
