# Supplementary material for: Arabidopsis Plastid-RNA Polymerase RPOTp Is Involved in Abiotic Stress Tolerance
Source: Plants (Basel). 2020 Jul 2;9(7):834. doi: 10.3390/plants9070834 (PMC7412009; doi:10.3390/plants9070834)
Supplement: Supplementary file 1 [file plants-09-00834-s001.zip › plants-847469 supplementary/Table S3.docx]

**Table S3.** Primers used in this work

| Purpose | Gene | Primer names  (F: forward/R: reverse) | Sequences (5’→ 3’) |
| --- | --- | --- | --- |
| qRT-PCR | *ACTIN2* | AT3G18780-QF | GCACCCTGTTCTTCTTACCG |
|  |  | AT3G18780-QR | AACCCTCGTAGATTGGCACA |
|  | *AOX1A* | AOX1A-QF | GTTCTTCCAGAGGAGATATGGA |
|  |  | AOX1A-QR | TCCATCCTCCACTTTGCTCAAA |
|  | *COR15B* | COR15B-QF | CAACGAAGCCACAAAGAAAGCT |
|  |  | COR15B-QR | CTCAGTCGCAGTTTCATTGGC |
|  | *LHCB1* | LHCB1s | ATGGCCGCCTCAACAATGG |
|  |  | LHCB1a | CGGTAAGGTAGCTGGGTGAC |
|  | *mTERF5* | AT4G14605-QF | TACCTCGTAGGAAGAGAGCTTA |
|  |  | AT4G14605-QR | GGATCAGGGAAGCTCACAACA |
|  | *mTERF9* | AT5G55580-QF | GGTCGAGGAAAGATGAGTTCGA |
|  |  | AT5G55580-QR | CTTAAGTAATCCAGAAGTGGTACA |
|  | *RD29A* | RD29A-QF | ATGCACCAGGCGTAACAGG |
|  |  | RD29A-QR | TCTTGTACTGGTACAGATT |
|  | *RPOTm* | AT1G68990-QF | GGAGCCAGTATATGAGGCTTTA |
|  |  | AT1G68990-QR | CTCTTCTGGAATGGGTACATCTT |
|  | *RPOTmp* | AT5G15700-QF | GGTAGCGAAAGGAAGCATGAAT |
|  |  | AT5G15700-QF | GCTTGGCTCCATGAGTTTTCAT |
|  | *RPOTp/SCA3* | AT2G24120-QF | CTTGGTGATTGTGCAAAGATAATT |
|  |  | AT2G24120-QR | GGGAGGAAATGCAGTTCTTTGTT |
|  | *psaA^1^* | ATCG00350-QF | GGCACAAGCATCTCAGGTAA |
|  |  | ATCG00350-QR | TAACCACGCCCGCTGAATAG |
|  | *psaB^1^* | ATCG00340-QF | CTCAGGACCCCACTACTCGT |
|  |  | ATCG00340-QR | TTGCCCGAAATGAGAAGC |
|  | *psbA* | ATCG00020-QF | GAGCAGCAATGAATGCGATA |
|  |  | ATCG00020-QR | CCTATGGGGTCGCTTCTGTA |
|  | *clpP* | ATCG00670-QF | GGTTGACATATACAACCGACTTT |
|  |  | ATCG00670-QR | CCATCCACCAGGAGAGTTTATA |
|  | *rps18* | ATCG00650-QF | GACGGGTGAATAGAGTGACTTT |
|  |  | ATCG00650-QR | GGAGTCGACTCACTTCTTTCAA |
|  | *rpoA^1^* | ATCG00740-QF | CAGGCATGAATACAGCATCG |
|  |  | ATCG00740-QR | CCCGCTGTGGAAATCATTG |
|  | *rpoB* | ATCG00190-QF | GCAGGTTAGAATTAGAGATTGATA |
|  |  | ATCG00190-QR | GGGTAGCAAACATTCTCTAGAAT |
|  | *rpoC1* | ATCG00180-QF | GCCTAGTATACTGCGATTTTTC |
|  |  | ATCG00180-QR | CTCGATTTCGAAATATATCGAAAC |
|  | *accD* | ATCG00500-QF | GCTAAGTAAAGCAATGGATAGTTT |
|  |  | ATCG00500-QR | CGAATGTCCTTGGAGCTAACTAA |

^1^Danilova et al., (2019).
