# Supplementary figures and images for: Arabidopsis Plastid-RNA Polymerase RPOTp Is Involved in Abiotic Stress Tolerance
Source: Plants (Basel). 2020 Jul 2;9(7):834. doi: 10.3390/plants9070834 (PMC7412009; doi:10.3390/plants9070834)

Figure S1

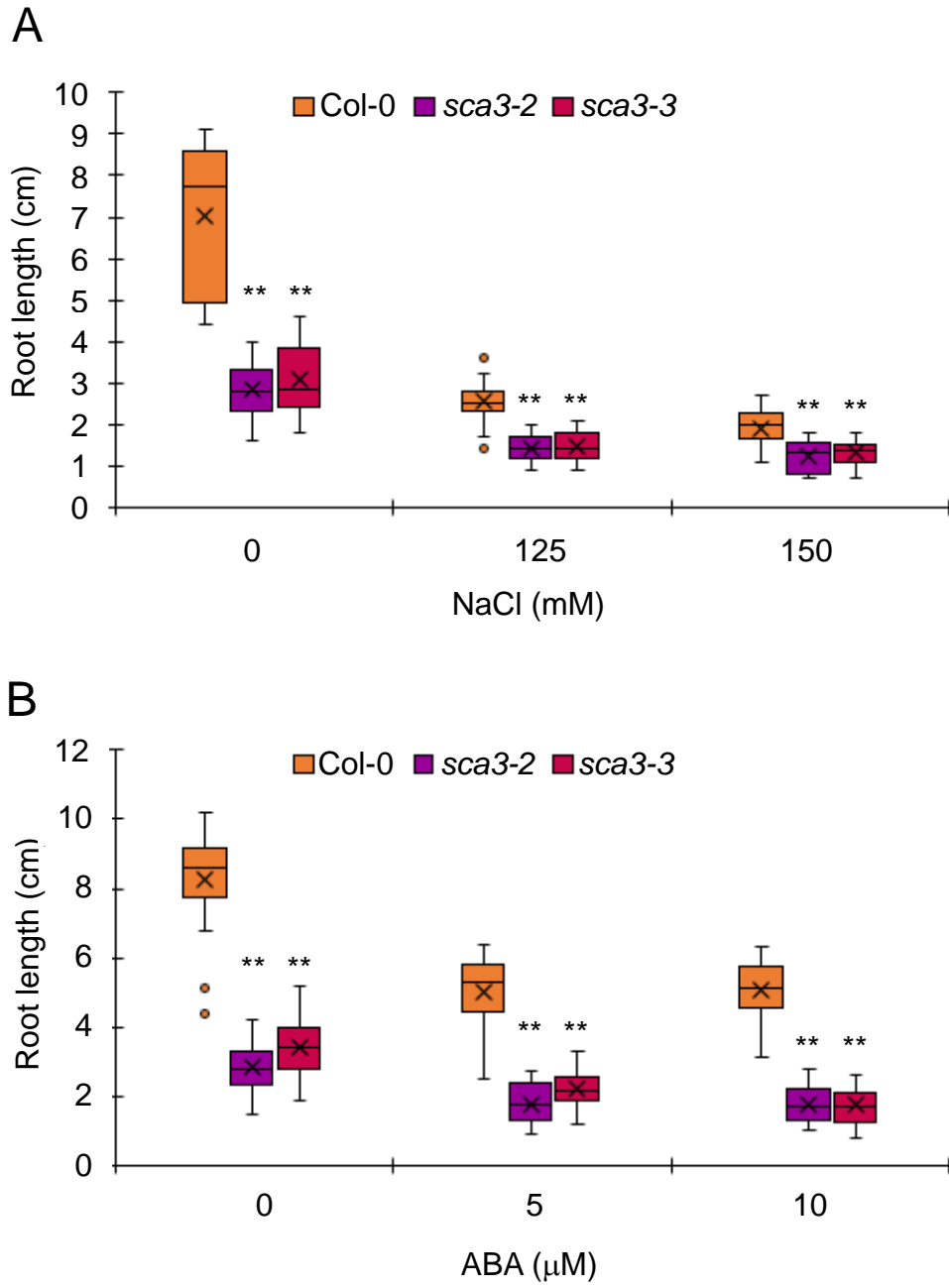

Supplement: Supplementary file 1 [file plants-09-00834-s001.zip › plants-847469 supplementary/Supplemental Fig 1.pdf]
